# The status and interpretation of neuropsychological evidence: Commentary on ‘No evidence yet for functional independence of verbal short‐term memory and long‐term verbal knowledge’ by Majerus, Cowan and Oberauer (2026)

**DOI:** 10.1111/jnp.70035

**Published:** 2026-02-05

**Authors:** Robert H. Logie

**Affiliations:** ^1^ University of Edinburgh Edinburgh UK

The debate as to whether short‐term and long‐term verbal memory and processing are distinct has a very long history, lasting over six decades in various forms, based on empirical research with healthy individuals and with brain damaged individuals who have contrasting, specific cognitive impairments. The target article by Bormann et al. (2026) focuses on evidence that the ability to retain a short verbal sequence in sequential order can be impaired, with intact functioning of long‐term learning and intact access to stored knowledge as well as to other aspects of language processing. However, the short commentary from Majerus et al. (2026) does not actually challenge that distinction. The title of their commentary argues that there is ‘No evidence yet for functional independence of verbal short‐term memory and long‐term verbal knowledge’. However, Bormann et al. and other researchers (e.g. Shallice & Papagno, 2019; Vallar, 2019) do not claim that STM patients do not use long‐term verbal knowledge, so the commentary does not address or critique the target article. As Majerus et al. point out, and Bormann et al. argue in the introduction and discussion of their article, word knowledge is required to identify that the stimuli are digits, words, letters etc., and individuals with impairments in retaining short verbal sequences in serial order have no difficulty with identifying phonology and articulation associated with the stimuli. All of their participants showed intact ability to repeat single words, even if other aspects of word processing were impaired.

Bormann et al. focus specifically on two previous speculative critiques that individuals with impaired immediate verbal serial ordered recall are rare and might have atypical language organisation and that such individuals might have subtle, hitherto undetected impairments of word perception and production. Majerus et al. do not acknowledge that the data presented by Bormann et al. are not consistent with these speculations but appear to focus on claims that Bormann et al. do not make.

The evidence presented by Bormann et al. (2026) may be described as an impairment in one specific aspect of language processing or as an impairment in temporary memory for a verbal sequence. However, these alternatives could be seen as simply relabelling from different perspectives rather than arguing for fundamentally different theoretical positions (for more extensive discussions see Logie, 2019, 2023). The task of immediate serial ordered verbal recall can be described as a memory function because it requires the participant to generate a response after the stimulus is no longer present, or it can be described as a language function because it involves linguistic material and processing. Which description is used depends on whether the focus is on understanding memory or on understanding language processing. As Bormann et al. note, clearly memory and language interact: some aspects of language are inevitably required for the verbal memory task, and retention (i.e. memory) of verbal material after stimulus offset is required for both post‐stimulus language processing and for the subsequent response. Even their patient HS, with language processing impairments and better verbal span than the other patients, had intact ability for word repetition, suggesting intact access to stored knowledge about phonological and articulatory codes that could support her span performance. Is there really a fundamental debate here, or is this simply a difference in preferred research perspective?

Cowan et al. (2014) suggested a distinction between ‘central and peripheral components of working memory storage’. In that context, the verbal STM patients described by Bormann et al. may have an impairment in a verbal peripheral component for retaining verbal serial order but not in a central component. It would be an interesting test of computational implementations of alternative theoretical positions to explore whether patterns of impairment and sparing observed in individuals with impairments, such as those described by Bormann et al., can be observed with simulated lesions in the models.

I have enormous respect for the authors of this commentary, even if we disagree about the status and importance, as well as the interpretation of data from studies of individuals with acquired brain damage. However, the Majerus et al. commentary addresses claims that are not made by Bormann et al. and unfortunately serves to perpetuate polemic rather than help with understanding the nature of the impairments from which these patients suffer, or with understanding the implications of the neuropsychological data for healthy memory and language function.

## CONFLICT OF INTEREST STATEMENT

There are no conflicts of interest.

## Data Availability

Data sharing is not applicable to this article as no datasets were generated or analysed during the current study.
